# Reproducibility of the STARD checklist: an instrument to assess the quality of reporting of diagnostic accuracy studies

**DOI:** 10.1186/1471-2288-6-12

**Published:** 2006-03-15

**Authors:** Nynke Smidt, Anne WS Rutjes, Daniëlle AWM van der Windt, Raymond WJG Ostelo, Patrick M Bossuyt, Johannes B Reitsma, Lex M Bouter, Henrica CW de Vet

**Affiliations:** 1 Institute for Research in Extramural Medicine, VU University Medical Center, Van der Boechorststraat 7, 1081 BT Amsterdam, The Netherlands; 2 Department of Clinical Epidemiology & Biostatistics, Academic Medical Center, University of Amsterdam, PO Box 22700, 1100 DE Amsterdam, The Netherlands

## Abstract

**Background:**

In January 2003, STAndards for the Reporting of Diagnostic accuracy studies (STARD) were published in a number of journals, to improve the quality of reporting in diagnostic accuracy studies. We designed a study to investigate the inter-assessment reproducibility, and intra- and inter-observer reproducibility of the items in the STARD statement.

**Methods:**

Thirty-two diagnostic accuracy studies published in 2000 in medical journals with an impact factor of at least 4 were included. Two reviewers independently evaluated the quality of reporting of these studies using the 25 items of the STARD statement. A consensus evaluation was obtained by discussing and resolving disagreements between reviewers. Almost two years later, the same studies were evaluated by the same reviewers. For each item, percentages agreement and Cohen's kappa between first and second consensus assessments (inter-assessment) were calculated. Intraclass Correlation coefficients (ICC) were calculated to evaluate its reliability.

**Results:**

The overall inter-assessment agreement for all items of the STARD statement was 85% (Cohen's kappa 0.70) and varied from 63% to 100% for individual items. The largest differences between the two assessments were found for the reporting of the rationale of the reference standard (kappa 0.37), number of included participants that underwent tests (kappa 0.28), distribution of the severity of the disease (kappa 0.23), a cross tabulation of the results of the index test by the results of the reference standard (kappa 0.33) and how indeterminate results, missing data and outliers were handled (kappa 0.25). Within and between reviewers, also large differences were observed for these items. The inter-assessment reliability of the STARD checklist was satisfactory (ICC = 0.79 [95% CI: 0.62 to 0.89]).

**Conclusion:**

Although the overall reproducibility of the quality of reporting on diagnostic accuracy studies using the STARD statement was found to be good, substantial disagreements were found for specific items. These disagreements were not so much caused by differences in interpretation of the items by the reviewers but rather by difficulties in assessing the reporting of these items due to lack of clarity within the articles. Including a flow diagram in all reports on diagnostic accuracy studies would be very helpful in reducing confusion between readers and among reviewers.

## Background

Over the past ten years, awareness of the importance of the quality of reporting of research articles has increased. Many systematic reviews have emphasized the poor quality of research reports. [1-3] Important aspects of design and results, such as a patient flow and adverse events, are often lacking in primary research articles. [1,3-7] To remedy this, guidelines have been developed to improve the reporting of randomised controlled trials (CONSORT), diagnostics accuracy studies (STARD), systematic reviews of randomised controlled trials (QUOROM) and observational studies (MOOSE). [8-14] Recently, also a checklist for STrengthening the Reporting of OBservational studies in Epidemiology (STROBE) has been developed. [15]

After the publication of the CONSORT statement in 1996, Moher et al. evaluated the quality of reporting in 211 randomised controlled trails published in *British Medical Journal, the Journal of the American Medical Association*, the *Lancet*, and the *New England Journal of Medicine *by using the CONSORT checklist. [16] They concluded that the use of the CONSORT statement was associated with improvements in the quality of reporting of randomised controlled trials [16]. The presentation of a flow diagram also improved the quality of reporting of randomised controlled trials. [17]

In January 2003, guidelines for the reporting of studies on diagnostic accuracy (STARD statement) were published simultaneously in eight medical journals *(Radiology, American Journal of Clinical Pathology, Annals of Internal Medicine, British Medical Journal, Clinical Biochemistry, Clinical Chemistry, Clinical Chemistry of Laboratory Medicine, and Lancet)*. The STARD statement contains a list of 25 items and encourages the use of a flow diagram to represent the design of the study and the flow of patients through the study. [11,12] These items were identified by an extensive search in the literature by the STARD steering committee and subsequently reviewed during a two-day consensus meeting with researchers, editors, and members of professional organizations. [11,12] Although the STARD statement was piloted for a number of months, there was no formal evaluation of its applicability.

Recently, we have evaluated the quality of reporting of 124 diagnostic accuracy studies published in 2000 (Pre-STARD evaluation) in 12 medical journals, using the items of the STARD statement. [18] We concluded that the quality of reporting in diagnostic accuracy articles is less than optimal, even in journals with an impact factor above 4. [18]

In order to evaluate the improvement of the quality of reporting of diagnostic accuracy studies published after the STARD statement, knowledge of the reproducibility of the assessment of the STARD checklist is needed. In addition, reproducibility of the individual STARD items gives input to the potential future adaptation or rewording of the STARD statement. Therefore, a reproducibility study was carried out within the evaluation study of the STARD statement. Our objective was to investigate the inter-assessment reproducibility of evaluating the quality of reporting of diagnostic accuracy studies published in 2000, using the items of the STARD statement. In addition, the intra- and inter-observer reproducibility was calculated to gain more insight into the sources of variation.

## Methods

### Selection of studies

Eligible papers for this reproducibility study were studies on diagnostic accuracy published in 2000 in one of the following 12 journals: Annals of Internal Medicine, Archives of Internal Medicine, Archives of Neurology, BMJ, Circulation, Clinical Chemistry, Gut, JAMA, Lancet, New England Journal of Medicine, Neurology, Radiology. These journals were selected based on the number of diagnostic accuracy studies published in 2000 and their impact factor (≥ 4). [18] The papers published in these journals had been included in a pre-STARD evaluation study, described elsewhere. [18]

An independent referee (ML, see acknowledgements) selected 32 out of 124 eligible diagnostic accuracy studies published in 2000. This referee had not been involved in any of the assessments. In making the selection, the referee took into account the quality of the reporting of the studies in order to ensure variability: half of the studies reported more than half of STARD items, the other half did not. Other selection criteria were a representative distribution of journals and an equal distribution of studies between the second reviewers.

## Study procedures

**Figure 1:**
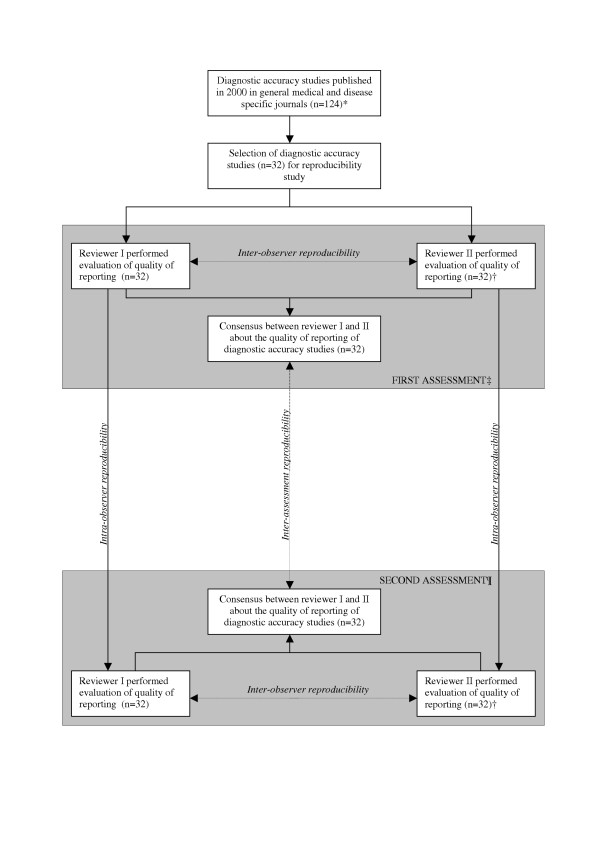
Overview of the design of the reproducibility study. * Papers were included in the pre-STARD evaluation, described elsewhere [18], † Four reviewers (AWSR, DAWMW, RWJGO, and HCWV) acted as second reviewer and each evaluated 8 articles. At the second assessment, the same reviewers evaluated the same studies, ‡ The first assessment was carried out together with the pre-STARD evaluation (March – May 2003), ¶The second assessment was carried out together with the post-STARD evaluation (January – March 2005).

An overview of the design of this reproducibility study is presented in Figure 1. The items of the STARD statement were used to assess the quality of reporting. The reviewers determined for each item whether it was adequately described in the text. Note that the reviewers were not evaluating the likelihood of bias, but only the quality of reporting.

During the first assessment, between March 2003 and May 2003 (Pre-STARD evaluation), two reviewers independently evaluated the quality of reporting in the included articles. One reviewer (NS) assessed all 32 articles and four other reviewers (AWSR, DAWMW, RWJGO, and HCWV) each evaluated a quarter of all the articles. Disagreements were discussed and resolved in a consensus meeting. If consensus could not be reached, a third reviewer made the final decision. All five reviewers are clinical epidemiologists and have experience in reviewing clinical articles.

During the second assessment (between January 2005 and March 2005), this procedure was repeated in the same sample of 32 studies. To enable assessment of intra-observer reproducibility, each study was assessed by the same two reviewers. The second assessment was carried out together with the Post-STARD evaluation; studies published in 2004 (data not shown).

### Statistical analysis

For each item in the STARD-statement, the total number of articles (%) reporting that item was calculated.

For the quantification of the reproducibility, three types of analysis were applied: Cohen's kappa statistics and the Bland and Altman method for assessing agreement and the Intraclass Correlation Coefficient (ICC) for the evaluation of reliability. Agreement provides insight into the distribution of differences between observers (or assessments), whereas reliability provides information on the ability of two observers (assessments) to differentiate between papers with high or poor quality of reporting.

To evaluate the inter-assessment agreement, we calculated percentages agreement between the first and second assessment and Cohen's kappa statistics for each item of the STARD-statement. A kappa value > 0 represents the observed agreement above chance agreement, where a value of 1 represents perfect agreement between reviewers or assessments. [19] To interpret the estimated kappa values, we used the classification suggested by Landis and Koch, indicating the strength of agreement: poor (kappa < 0.00), slight (kappa 0.00–0.20), fair (0.21–0.40), moderate (0.41–0.60), substantial (0.61–0.80), and almost perfect (0.81–1.00). [20]

For each assessment, we counted the total number of reported items in each article (range 0 to 25). As six items (items 8, 9, 10, 11, 13, 24) concern index tests as well as the reference standard, we counted the index test as 1/2 item and the reference standard as 1/2 item. The mean number of items and standard deviation were calculated for each assessment.

For the quantification of inter-assessment reproducibility of the total number of STARD items, we used the Bland and Altman method for evaluating agreement between the first and second assessment and the Intraclass Correlation Coefficients (ICC) for an evaluation of the reliability. [21]

The Bland and Altman method describes the distribution of differences in number of reported STARD items between the first and second assessment. Inter-assessment agreement was quantified by calculating the mean difference (*d*) of the number of STARD items reported between the first and second assessment and its standard deviation (SD) for this difference. The closer *d *is to zero and the smaller the SD of this difference, the better the inter-assessment agreement. Differences between the first and second assessment were plotted against the mean number of reported STARD items of the two assessments. This graph shows the size, direction, and range of the differences and shows whether differences between assessments are consistent across the range of total number of STARD items reported. The 95% limits of agreement were calculated as the mean difference between the first and second assessment ± 1.96 SD of the differences, indicating the total error (bias and random error together). The presence of bias between both assessments was estimated by calculating the 95% confidence interval (95% CI) for *d*. The 95% confidence interval for *d *was calculated as *d *± 1.96 * [SDdiff/√ n], where n is the number of articles included in this study. If zero lies outside the 95% CI, systematic differences (bias) between the observers exist. [21]

When trying to determine whether introduction of the STARD statement has improved the quality of reporting, this change must overcome the measurement error, ie. be larger than the smallest detectable difference (SDD). The SDD was calculated as 1.96 * SD_diff_, in which SD_diff _represents the standard deviation of the difference between the measurement values of the first and second assessment. [22]

The ICC provides information on the ability to differentiate between articles with regard to quality of reporting (reliability). The ICC was defined as the ratio of variance in quality of reporting of articles (article variability) over the total variance (including article variability, assessment variability, random error variability). The ICC takes values in the range of 0 (no reliability) to 1 (perfect reliability). An ICC of less than 0.75 was considered unsatisfactory. [23] Two-way random effect models were used to calculate ICCs according to Fleiss. [19] Data-entry and statistical analysis using SPSS for Windows (Release 11.0.1, 2001) were done by NS.

In addition, for each item of the STARD statement, the intra-observer agreement before consensus was evaluated for the first (NS) and second reviewer separately. Note that, the second review was carried out by four different reviewers (AWSR, DAWMW, RWJGO, HCWV), each reviewer evaluating eight of the 32 articles.

Furthermore, the inter-observer agreement (first versus second reviewer) was calculated for both assessments to study whether reviewers interpreted items differently.

The a priori calculation of the number of articles to be included in the evaluation was based on a paired t-test for a difference of one item between the means of two assessments, expecting a standard deviation of the difference between assessments of 2 items. Using a two-tailed α of 0.05, a total of 32 articles would be required to achieve a power of 80%.

## Results

### Study characteristics

The 32 diagnostic accuracy studies published in 2000 included 25 cohort and 7 case control studies. Ten articles were published in a general medical journal (Annals of Internal Medicine (n = 3), Archives of Internal Medicine (n = 1), British Medical Journal (n = 2), Journal of the American Medical Association (n = 4)) and 22 in disease or discipline specific journals (Archives of Neurology (n = 2), Circulation (n = 4), Clinical Chemistry (n = 4), Gut (n = 4), Neurology (n = 4) and Radiology (n = 4)). In most studies, the diagnostic accuracy of laboratory tests (n = 14) or imaging tests (n = 17) was examined. In only one study, the diagnostic value of history taking and physical examination was investigated. There was a large variety in the target conditions described in the studies, which included Pompe disease, deep vein thrombosis, acute stroke, colorectal neoplasia, colbalamin deficiency, cervical cancer, extra temporal lobe epilepsy in children, skeletal dysplasia, pulmonary embolism, and obstructive airways diseases.

### Inter-assessment reproducibility

#### Reporting of STARD items

The results of the quality of reporting for both assessments and the percentage of agreement for each item of the STARD statement between the first and second assessment are presented in Table 1. There was a large variation in the quality of reporting of the items of the STARD statement. Some items (item 25) were reported by almost all articles, whereas other items (item 1, item 13b, 24b) were rarely positively evaluated.

**Table 1:** Number of articles reported the items of the STARD statement at the first and second assessment and for each item the percentage agreement between the two assessments and kappa statistics of the two assessments.*

		First assessment (n = 32)	Second assessment (n = 32)	Inter-assessment agreement	Cohen's kappa
Item		n (%)	n (%)	n (%)	

*Title/abstract/keywords*					
1	Identify the article as a study of diagnostic accuracy (recommend MeSH heading 'sensitivity and specificity').	3 (9)	1 (3)	94	0.48

*Introduction*					

2	State the research questions or study aims, such as estimating diagnostic accuracy or comparing accuracy between tests or across participant groups.	27 (84)	31 (97)	88	0.30
*Methods*					

3	The study population: The inclusion and exclusion criteria, setting and locations where data were collected.	17 (53)	10 (31)	78	0.57
4	Participant recruitment: Was recruitment based on presenting symptoms, results from previous tests, or the fact that the participants had received the index tests or the reference standard?	28 (88)	32 (100)	88	NA
5	Participant sampling: Was the study population a consecutive series of participants defined by the selection criteria in item 3 and 4? If not, specify how participants were further selected.	20 (63)	25 (78)	84	0.64
6	Data collection: Was data collection planned before the index test and reference standard were performed (prospective study) or after (retrospective study)?	25 (78)	26 (81)	84	0.52
7	The reference standard and its rationale.	14 (44)	14 (44)	69	0.37
8	Technical specifications of material and methods involved including how and when measurements were taken, and/or cite references for a) index tests and b) reference standard.	28 (88) 19 (59)	29 (91) 19 (59)	97 75	0.84 0.48
9	Definition of and rationale for the units, cut-offs and/or categories of the results of the a) index tests and the b) reference standard.	26 (81) 20 (63)	26 (81) 23 (72)	81 78	0.39 0.51
10	The number, training and expertise of the persons executing and reading the a) index tests and the b) reference standard.	13 (41) 11 (34)	13 (41) 10 (31)	94 84	0.87 0.65
11	Whether or not the readers of the a) index tests and b) reference standard were blind (masked) to the results of the other test and describe any other clinical information available to the readers.	9 (28) 8 (25)	10 (31) 12 (38)	84 88	0.63 0.71
12	Methods for calculating or comparing measures of diagnostic accuracy, and the statistical methods used to quantify uncertainty (e.g. 95% confidence intervals).	4 (13)	4 (13)	94	0.71
13	Methods for calculating test reproducibility, if done a) for the index test b) for the reference standard.	4 (13) 2 (6)	8 (25) 2 (6)	88 94	0.60 0.47

*Results*					

14	When study was performed, including beginning and end dates of recruitment.	17 (53)	17 (53)	100	1.00
15	Clinical and demographic characteristics of the study population (at least information on age, gender, spectrum of presenting symptoms).	14 (44)	16 (50)	81	0.63
16	The number of participants satisfying the criteria for inclusion who did or did not undergo the index tests and/or the reference standard, describe why participants failed to undergo either test (a flow diagram is strongly recommended).	20 (63)	19 (59)	66	0.28
17	Time-interval between the index tests and the reference standard, and any treatment administered in between.	7 (22)	9 (28)	81	0.50
18	Distribution of severity of disease (define criteria) in those with the target condition, other diagnoses in participants without the target condition.	9 (28)	15 (47)	63	0.23
19	A cross tabulation of the results of the index tests (including indeterminate and missing results) by the results of the reference standard, for continuous results, the distribution of the test results by the results of the reference standard.	24 (75)	24 (75)	75	0.33
20	Any adverse events from performing the index tests or the reference standard.	5 (16)	5 (16)	100	1.00
21	Estimates of diagnostic accuracy and measures of statistical uncertainty (e.g. 95% confidence intervals).	13 (41)	14 (44)	91	0.81
22	How indeterminate results, missing data and outliers of the index tests were handled.	20 (63)	21 (66)	66	0.25
23	Estimates of variability of diagnostic accuracy between subgroups of participants, readers or centers, if done.	14 (44)	17 (53)	91	0.81
24	Estimates of test reproducibility, if done. a) index test b) reference standard	8 (25) 1 (3)	10 (31) 2 (6)	81 97	0.54 0.65

*Discussion*					

25	Discuss the clinical applicability of the study findings.	31 (97)	31 (97)	94	-0.032

* Data extraction form for assessing the 25 items of the STARD statement and the references of the 32 included articles are available on request of the first author; NA = not able to calculate.

The overall inter-assessment agreement for all items of the STARD statement was 85% (Cohen's kappa 0.70), and varied from 63% (item 18) to 100% (items 14, 20 and flow diagram). The largest differences (≤ 75 % agreement and Cohen's kappa ≤ 0.40) between the two assessments were found for the description of the rationale of the reference standard (item 7), the number of included participants that underwent tests and description why participants failed to undergo either test (item 16), the distribution of the severity of the disease in those with the target condition and other diagnosis in participants without the target condition (item 18), a cross tabulation of the results of the index test by the results of the reference standard (item 19), and the reporting how indeterminate results, missing data and outliers of the index test were handled (item 22). In accordance with the inter-assessment agreement for the above-mentioned items, a Cohen's kappa between 0.21 and 0.40 was observed, which means 'fair' according the classification of Landis and Koch. [20] In contrast, the inter-assessment agreement for the discussion of the clinical applicability of study findings (item 25) was quite high (94%), but the kappa statistics was poor (< 0.00). This low kappa can be explained by the high prevalence (97%) of reporting this item in the articles, indicating that it is an item that can be agreed on easily. [24]

#### Reporting of number of STARD items per article

Table 2 summarizes the results of the inter-assessment agreement of the total number of reported STARD items. The mean number of reported items was 12.3 items per article. None of the articles included a flow diagram.

**Table 2:** Inter-assessment agreement: mean of first assessment and second assessment of the quality of reporting of diagnostic accuracy studies (n = 32), followed by mean differences between the two assessments, 95% limits of agreement, and smallest detectable difference (SDD).

Outcome measure	First assessment (A)			Second assessment (B)			Difference B – A Mean_diffAB _ (SD_diffAB_)	95% Limits of Agreement*	SDD†	Systematic differences (95%CI)‡
	Mean_A _(SD_A_)	RangeA	Mean_B _(SD_B_)	Range_B_				
Number of reported STARD items (0–25)	12.08 (3.9)	3.5 – 19	12.47 (3.4)	7 – 19	0.39 (2.4)	-4.27, 5.05	4.66	0.39 (-0.4, 1.2)

SD = Standard Deviation, * 95% Limits of Agreement: mean_(*diffAB*)± _1.96SD_diffAB_, † SDD = Smallest Detectable Difference (SDD = 1.96 * SD_diffAB_); ‡ systematic difference (bias) = Mean_diffAB_± 1.96 * SD_diffAB_/√ n; 95%CI = 95% confidence intervals.

**Figure 2:**
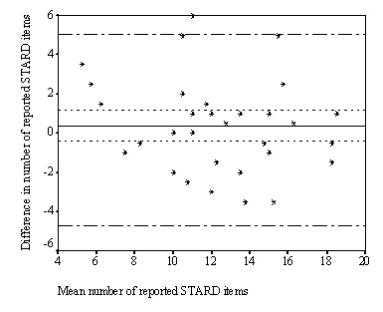
Differences between first and second assessment for each article (n = 32), plotted against the mean value of both assessments for the total number of reported STARD items. Solid line: mean difference (0.39) between the two assessments, short striped lines: 95% Confidence Intervals (-0.4, 1.2) of systematic differences, long striped lines: 95% limits of agreement (-4.3, 5.0).

Figure 2 shows the Bland and Altman plot with differences between the two assessments on the y-axis and the mean number of reported STARD items of the two assessments on the x-axis. The mean number of STARD items reported varied from 5.3 to 18.5 items. We found a small but non-significant difference between the first and the second assessment of 0.39 items (95%CI -0.4 to 1.2). In 17 articles (53%), the total number of reported STARD items was slightly higher at the second assessment than the first assessment, while this number was similar in three articles (9%). The smallest detectable difference was 4.7, which means that the quality of reporting of an individual study published after the introduction of the STARD statement should report a least 4.7 items more than a study published before the introduction of the STARD statement, to overcome measurement error. When sample sizes increase, smaller difference can be detected as SDD on group level equals SDD individual level/√n.

The inter-assessment reliability of the total number of reported STARD items was satisfactory (ICC = 0.79 [95% CI = 0.62, 0.89]). The variance between the 32 articles, two assessments and the random error were 10.7, -0.01 and 2.8, respectively.

For subgroup analysis on design of the studies, numbers were too small.

### Intra-observer agreement

For the first and second reviewer, the intra-observer agreement for each item of the STARD statement are calculated and presented in Additional file 1. For the first reviewer, the intra-observer agreement for all items of the STARD statement was 83% (Cohen's kappa 0.66) and for the second reviewer 82% (Cohen's kappa 0.64). The intra-observer agreement varied from 56% (item 18) to 100% (item 14). Low intra-observer agreement (≤ 75% agreement for both observers) was found for item 7, item 16, item 18, item 19, item 22 and item 23). Except for item 23, these are the same items for which we observed the highest number of disagreements in the inter-assessment analysis.

### Inter-observer agreement

For the first and second assessment, the inter-observer agreement for each item of the STARD statement are calculated and presented in Additional file 1. Inter-observer agreement for all items of the STARD statement was 81% for the first assessment (Cohen's kappa 0.61) as well as for the second assessment (Cohen's kappa 0.63). Inter-observer agreement varied from 50% (item 18) to 100% (item 14, 20, and 24b). At both assessments, most disagreements (≤ 75% agreement) between reviewers were found for item 6, item 7, item 11b, item 15, item 16, item 18, item 19, and item 22.

## Discussion

The overall reproducibility of the assessment of the quality of reporting on diagnostic accuracy studies published in 12 medical journals with a high impact factor, using the STARD statement, was found to be good with a Cohen's kappa statistic of 0.70 and ICC of 0.79. Substantial disagreement was found for some items, including (a) the reporting of the rationale of the reference standard, (b) the number of included participants that undergo the index tests and/or the reference standard and description why participants failed to undergo either test, (c) the distribution of the severity of the disease in those with the target condition and other diagnosis in participants without the target condition, (d) a cross tabulation of the results of the index test by the results of the reference standard, and (e) how indeterminate results, missing data and outliers of the index test were handled. The results of the intra-observer and inter-observer reproducibility showed low observer agreements (≤ 75%) for the same items, indicating that reviewers had difficulties with the assessment of these items. If we had found high intra-observer but low inter-observer reproducibility, this would have pointed at different interpretation of the items. Therefore, these disagreements in our study were probably not caused by differences in interpretation of the items, but rather by difficulties in assessing the reporting of these items in the articles.

Stengel and colleagues found similar results in 62 diagnostic accuracy studies on ultrasonography for trauma. The inter-observer agreement of assessment of STARD items ranged from poor for specification of the number of patients who dropped out (58%) to almost perfect for the specification of the selection criteria (98%). [25]

Although four reviewers acted as second reviewer, we decided, based on the small number of studies assessed by these four reviewers, to ignore differences in scoring between the four reviewers and not calculate stratified reproducibility statistics. As the agreements of the reviewers with the first reviewer were comparable, this is unlikely to have influenced our results.

The presentation of a flow diagram, presenting the design of the study and the flow of patients through the study, would be helpful in improving the quality of reporting of diagnostic accuracy studies, as a flow diagram explicitly clarifies items that appeared to be difficult to assess. The optimal flow diagram presents the target population (setting, location and characteristics of potentially eligible persons and represents the individuals to whom the results are expected to apply), eligible population (proportion of potential participants who undergo screening and are eligible to enroll), and the actual research population (eligible patients who are willing to participate; informed consent). The number of participants who did not satisfy the eligibility criteria, reasons for exclusion, number of participants who failed to receive one of tests and the results of the index tests (including indeterminate and missing results) by the results of the reference standard representing the true positives, true negatives, false positives and false negatives can easily be reported in a flow diagram. The intra- and inter-observer disagreements regarding item 16 and 22 were caused due confusion regarding the final research population. If in a study, patients were excluded from the analysis because they did not receive one of the tests (missing) and the reasons for exclusion were not specified, it was unclear whether these patients belonged to the actual research population or not.

A large variety in diseases and tests were included in our study. This was a result of our decision to select all diagnostic accuracy studies published in 2000 in general medical journals and discipline specific journals. Although a pilot evaluation of the quality of reporting was carried out among all reviewers, no additional criteria were defined for specification of the severity of all diseases (item 18) described in the studies. Evaluating the reporting of this item was affected by the differences in the subjective judgment of the reviewers. A similar observation was made reporting the rationale of the reference standard. More detailed specification of these items is possible if the STARD statement is used to evaluate papers about a specific subject. Items such as the recruitment period, adverse events of the tests and presentation of a flow diagram are less susceptible to subjective judgment of the reviewers, resulting in higher inter-assessment, intra-observer and inter-observer agreement.

High inter-assessment agreement was observed for the reporting of those items that are associated with biased estimates of diagnostic accuracy, such as the blinding of the readers of the index and reference test to the results of the other test and the clinical information and the description of the study population. [26-28] Furthermore, the results of this reproducibility study indicates the importance of the need of at least two reviewers who independently assess the quality of reporting of diagnostic accuracy studies.

We recommend that the STARD steering committee should discuss the results of this reproducibility study and decide whether certain items of the STARD statement should be more clarified in the statement as these items cause difficulties in the assessment of the quality of reporting. To our opinion, including a flow diagram in all reports on diagnostic accuracy studies would be very helpful for both readers and reviewers.

As this reproducibility study did yield important information for the applicability of the STARD statement, this could also be the case for other guidelines such as CONSORT, QUOROM, MOOSE and STROBE.

## Conclusion

In summary, this study shows that agreement in evaluating the reported STARD items is satisfactory. The evaluation of the quality of reporting of diagnostic accuracy studies is not trivial, as the assessment of some STARD items are potentially affected by the subjective judgment of the reviewers. In order to improve an unequivocal interpretation of the study design and the flow of patients through the study, a flow diagram is an indispensable tool for diagnostic accuracy studies.

## Competing interests

PMB, HCWV and JBR are members of the STARD steering commitee. All other authors declare that they have no competing interests.

## Authors' contributions

NS and HCWV were responsible for the conception and design of the study. NS, AWSR, HCWV, RWJGO, and DAWMW participated in data-extraction. NS performed the analysis and drafted the article. All authors contributed to the interpretation of the data, and all authors read and approved the final manuscript.

## Pre-publication history

The pre-publication history for this paper can be accessed here:



## Supplementary Material

Additional file 1 Click here for file
