# Supplementary material for: Reproducibility of the STARD checklist: an instrument to assess the quality of reporting of diagnostic accuracy studies
Source: BMC Med Res Methodol. 2006 Mar 15;6:12. doi: 10.1186/1471-2288-6-12 (PMC1522016; doi:10.1186/1471-2288-6-12)
Supplement: Additional file 1 [file 1471-2288-6-12-S1.doc]

Appendix I. Number of articles that reported the STARD items according to both reviewers at the first and second assessment and for each item the percentage intra-observer agreement between the two assessments and kappa statistics and the inter-observer agreement and kappa statistics at the first and second assessment.

|  | Intra-observer reproducibility | | | | | | | | | | | | Inter-observer reproducibility | | | |
| --- | --- | --- | --- | --- | --- | --- | --- | --- | --- | --- | --- | --- | --- | --- | --- | --- |
|  | **Reviewer I** | | | | | | **Reviewer II** | | | | | |  | | | |
|  | First assessment | | Second assessment | | Intra-observer agreement | | First  assessment | | Second assessment | | Intra-observer  agreement | | Inter-observer agreement  (first assessment) | | Inter-observer agreement  (second assessment) | |
| Items | n | (%) | n | (%) | Agreement  % | Cohen’s kappa | n | (%) | n | (%) | Agreement % | Cohen’s kappa | Agreement % | Cohen’s kappa | Agreement  % | Cohen’s kappa |
| 1 | 3 | (9) | 2 | (6) | 91 % | 0.35 | 5 | (16) | 1 | (3) | 88 % | 0.30 | 94 % | 0.72 | 97 % | 0.65 |
| 2 | 29 | (91) | 32 | (100) | 91 % | NA | 26 | (81) | 28 | (88) | 94 % | 0.77 | 84 % | 0.37 | 88 % | NA |
| 3 | 16 | (50) | 9 | (28) | 78 % | 0.56 | 12 | (38) | 8 | (25) | 69 % | 0.29 | 81 % | 0.63 | 78 % | 0.44 |
| 4 | 29 | (91) | 31 | (97) | 88 % | -0.05 | 27 | (84) | 30 | (94) | 84 % | 0.22 | 81 % | 0.15 | 91 % | -0.04 |
| 5 | 18 | (56) | 24 | (75) | 81 % | 0.60 | 23 | (72) | 25 | (78) | 88 % | 0.67 | 84 % | 0.67 | 84 % | 0.57 |
| 6 | 21 | (66) | 22 | (69) | 78 % | 0.50 | 26 | (81) | 28 | (88) | 75 % | 0.06 | 72 % | 0.30 | 69 % | 0.13 |
| 7 | 12 | (38) | 12 | (38) | 69 % | 0.33 | 16 | (50) | 14 | (44) | 75 % | 0.50 | 63 % | 0.25 | 69 % | 0.36 |
| 8a | 29 | (91) | 31 | (97) | 88 % | -0.05 | 28 | (88) | 27 | (84) | 91 % | 0.61 | 91 % | 0.52 | 88 % | 0.30 |
| 8b | 19 | (59) | 22 | (69) | 84 % | 0.66 | 19 | (59) | 15 | (47) | 69 % | 0.38 | 69 % | 0.35 | 78 % | 0.57 |
| 9a | 24 | (75) | 26 | (81) | 81 % | 0.46 | 25 | (78) | 26 | (81) | 78 % | 0.33 | 84 % | 0.57 | 75 % | 0.18 |
| 9b | 19 | (59) | 20 | (63) | 78 % | 0.54 | 18 | (56) | 22 | (69) | 81 % | 0.61 | 66 % | 0.30 | 81 % | 0.59 |
| 10a | 14 | (44) | 15 | (47) | 91 % | 0.81 | 10 | (31) | 11 | (34) | 91 % | 0.79 | 88 % | 0.74 | 88 % | 0.75 |
| 10b | 12 | (38) | 11 | (34) | 91 % | 0.80 | 9 | (28) | 6 | (19) | 78 % | 0.40 | 84 % | 0.65 | 84 % | 0.61 |
| 11a | 6 | (19) | 11 | (34) | 78 % | 0.46 | 12 | (38) | 9 | (28) | 84 % | 0.65 | 75 % | 0.41 | 88 % | 0.71 |
| 11b | 4 | (13) | 7 | (22) | 84 % | 0.46 | 11 | (34) | 9 | (28) | 81 % | 0.57 | 72 % | 0.27 | 75 % | 0.34 |
| 12 | 6 | (19) | 6 | (19) | 81 % | 0.39 | 6 | (19) | 4 | (13) | 88 % | 0.53 | 88 % | 0.59 | 88 % | 0.53 |
| 13a | 5 | (16) | 9 | (28) | 88 % | 0.64 | 6 | (19) | 5 | (16) | 91 % | 0.67 | 78 % | 0.23 | 88 % | 0.64 |
| 13b | 2 | (6) | 3 | (9) | 97 % | 0.78 | 2 | (6) | 2 | (6) | 94 % | 0.47 | 94 % | 0.47 | 97 % | 0.78 |
| 14 | 17 | (53) | 17 | (53) | 100 % | 1.00 | 17 | (53) | 17 | (53) | 100 % | 1.00 | 100 % | 1.00 | 100 % | 1.00 |
| 15 | 18 | (56) | 18 | (56) | 81 % | 0.62 | 14 | (44) | 14 | (44) | 81 % | 0.62 | 63 % | 0.26 | 75 % | 0.51 |
| 16 | 14 | (44) | 21 | (66) | 59 % | 0.22 | 18 | (56) | 18 | (56) | 75 % | 0.49 | 63 % | 0.26 | 66 % | 0.29 |
| 17 | 8 | (25) | 8 | (25) | 81 % | 0.50 | 8 | (25) | 6 | (19) | 69 % | 0.09 | 88 % | 0.67 | 69 % | 0.09 |
| 18 | 10 | (31) | 14 | (44) | 75 % | 0.48 | 12 | (38) | 14 | (44) | 56 % | 0.10 | 63 % | 0.17 | 50 % | -0.02 |
| 19 | 26 | (81) | 25 | (78) | 66 % | -0.06 | 23 | (72) | 24 | (75) | 72 % | 0.28 | 59 % | -0.12 | 66 % | 0.04 |
| 20 | 4 | (13) | 5 | (16) | 97 % | 0.87 | 4 | (13) | 5 | (16) | 97 % | 0.87 | 94 % | 0.71 | 100 % | 1.00 |
| 21 | 13 | (41) | 13 | (41) | 88 % | 0.74 | 12 | (38) | 11 | (34) | 97 % | 0.93 | 91 % | 0.80 | 88 % | 0.73 |
| 22 | 17 | (53) | 22 | (69) | 66 % | 0.30 | 19 | (59) | 17 | (53) | 69 % | 0.37 | 63 % | 0.24 | 72 % | 0.42 |
| 23 | 16 | (50) | 16 | (50) | 75 % | 0.50 | 16 | (50) | 16 | (50) | 63 % | 0.25 | 81 % | 0.63 | 63 % | 0.25 |
| 24a | 9 | (28) | 11 | (34) | 88 % | 0.71 | 7 | (22) | 7 | (22) | 88 % | 0.63 | 94 % | 0.83 | 88 % | 0.70 |
| 24b | 1 | (3) | 1 | (3) | 94 % | -0.03 | 1 | (3) | 2 | (6) | 97 % | 0.65 | 100% | 1.00 | 97 % | 0.65 |
| 25 | 29 | (91) | 30 | (94) | 84 % | -0.08 | 31 | (97) | 31 | (97) | 94 % | -0.03 | 94 % | 0.48 | 91 % | -0.04 |

NA = not able to calculate.
